# Understanding the Factors Influencing the Leisure Tourism Behavior of Visually Impaired Travelers: An Empirical Study in China

**DOI:** 10.3389/fpsyg.2021.684285

**Published:** 2021-06-21

**Authors:** Guanghui Qiao, Junmiao Zhang, Anja Pabel, Nan Chen

**Affiliations:** ^1^School of Tourism and Urban-Rural Planning, Zhejiang Gongshang University, Hangzhou, China; ^2^Zheshang Research Institute, Zhejiang Gongshang University, Hangzhou, China; ^3^School of Business and Law, Central Queensland University, Cairns Campus, Rockhampton, QLD, Australia; ^4^Research Institute for Study Travel, Henan University, Kaifeng, China

**Keywords:** leisure tourism, tourism behavior, vision impaired tourists, China, empirical study

## Abstract

This study looks at the real-world problems which vision impaired individuals face when they travel. More specifically, this study aims to explore the main factors influencing the leisure tourism behavior of visually impaired individuals. Based on in-depth semi-structured interviews with 26 visually impaired respondents, this study identifies six main factors impacting on the leisure tourism behavior of visually impaired individuals including: tourism products and services, personal psychological factors, social support, community support, personal socio-economic factors, and barrier-free environments. Findings show that visually impaired travelers have strong requirements for auditory, tactile, and physical participation. Support factors such as travel companions/escorts, tour organizers specifically targeting their experiential offerings at the visually impaired, and an accessible environment are important considerations for visually impaired travelers. Findings also show that visually impaired individuals participate in leisure tourism to enhance their own abilities, relieve pressures on their families, break stereotypes associated with the visually impaired, and promote the need for greater tourism development specifically targeting visually impaired travelers. This study also proposes a theoretical model outlining the factors influencing leisure tourism of visually impaired people.

## Introduction

With the increase in leisure time and material wealth comes an enhanced awareness of leisure time as an important avenue for improving life happiness and realizing life meaning (Liu and Li, 2020). However, visually impaired individuals are constrained by their own physiological conditions, making it difficult for them to participate in leisure tourism. Because of their impaired visual function, they have long been imprisoned by the traditional opinion that tourism is about sightseeing which requires one to be able to see the sights (Xie, 2017). This is also the reason why we seldom see visually impaired people in scenes of daily life and leisure tourism (Zhang, 2019). Nonetheless, the number of visually impaired people is substantial, and there is potential for future tourism development to focus on the specific needs of this potential market segment. The World Health Organization estimates that 253 million people worldwide suffer from visual impairment, of whom about 36 million are completely blind, and about 217 million suffer from moderate to severe visual impairment (Bourne et al., 2017). As of 2016, there are ~17 million visually impaired people in China (Datagoo, 2020), and this figure is growing at a rate of about 450,000 individuals per year (ChinaIRN, 2019). Including relatives and friends of visually impaired individuals who often act as travel companions, the market volume is estimated to increase three to four times the absolute number of visually impaired people (China Disabled Persons' Federation, 2007). A survey conducted in China shows that more than 60% of disabled people have a strong desire to travel, and the proportion of disabled people who have a desire to travel is increasing (Liu, 2016). To promote tourism development targeting the visually impaired, it is important to gain their perspectives on the types of tourism experiences they prefer including appropriate supporting factors. There is a dearth of relevant tourism literature focusing on visually impaired people and their tourism related needs, and any barriers they face when accessing tourism experiences. Thus, there are significant benefits in considering the experiences of visually impaired individuals to build a theoretical model which identifies the factors influencing the tourism behavior of visually impaired people.

To fill this gap in the extant literature, this study aims to explore the factors influencing the leisure tourism behavior of visually impaired individuals, and to propose a theoretical model that is helpful in guiding future tourism development targeting the visually impaired. China has the largest number of visually impaired people in the world. Hence, this study focuses on Chinese visually impaired people, and attempts to uncover the “mysterious veil” of influencing factors that impact on their tourism behavior. It is also hoped that this study can deepen the public's understanding of visually impaired individuals and their needs for tourism experiences, and to provide guidelines for the creation of tourism products that better serve visually impaired people.

## Literature Review

### Leisure, Tourism, and Leisure Tourism for the Visually Impaired

Leisure is not only a kind of human behavior, but also a complex social phenomenon. No all-encompassing definition of leisure exists to fully describe its different states and meanings under different historical stages, cultural backgrounds, and social scenes (Guo, 2013). Existing interpretations of leisure focus on three perspectives: time, activity content, and psychology. From the perspective of time, a person's time can be divided into working time and free time (Ma, 2003). Apart from the time needed to maintain basic life and domestic chores, the rest of the free time is leisure time. Making full use of such time to carry out leisure activities is conducive to improving work efficiency and life satisfaction (Stebbins, 1996; Liu and Li, 2020). From the perspective of activity content, leisure is usually interpreted as non-work, non-obligatory and non-oppressive activities determined by personal preferences. Individuals may participate in activities that provide them with physical and mental pleasure, spiritual satisfaction, self-actualization, and the pursuit of leisure activities that cannot be obtained in their habitual activities (McLean, 2003). From the psychological perspective, “leisure” mainly emphasizes people's subjective attitudes and feelings. Leisure is regarded as a free, relaxed, sublimated, and unrestrained mental state, which is not only of pleasure value, but also of great significance to health, and the promotion of subjective well-being (Newman et al., 2014). Leisure can be divided into several types of activities (Guo, 2005). For example, tourism, sports, and cultural pursuits are all types of leisure (Liu, 2006). Traveling to unusual environments to obtain leisure tourism experiences has gradually become a major part of leisure (Cheng, 2006). Ideally, there should be no differences in the concept of leisure tourism for sighted individuals and for visually impaired individuals. However, the channels and ways for the visually impaired to participate in leisure tourism experiences are significantly different compared to sighted people (Kong and Loi, 2017). Currently, there is no special definition of leisure tourism for the visually impaired in the academic literature. In the context of this study, we propose the following definition of leisure tourism for visually impaired people: Tourism experiences specifically targeting vision impaired individuals who wish to travel. Such tourism experiences focus on sensory elements beyond sight and include aspects such as accommodation, restaurant, transportation and more accessible attractions and museums. Leisure tourism for visually impaired individuals can be divided into two categories: (1) family tourism for visually impaired people and (2) group tourism for visually impaired people. The first category recognizes that visually impaired individuals usually travel with their families and friends, which not only provides an opportunity for the visually impaired and their families and friends to have rest and relaxation together (Darcy, 2010), but also enables the visually impaired to have more harmonious social relations (Chang and Chen, 2012). The latter category of travel is mainly targeted at visually impaired people and includes a small number of service personnel. According to the nature of the group, this category can be further subdivided into non-profit tour groups and commercial tour groups. Non-profit tour groups for visually impaired people are usually organized by non-profit organizations or are organized by visually impaired people themselves (Meza et al., 2019). Alternatively, commercial tour groups for the visually impaired are organized by professional travel agencies. While it has previously been uncommon for visually impaired individuals to travel by themselves (Small et al., 2012), an increasing number of them are now traveling the world alone (Zhang, 2019).

### Factors Influencing Leisure Tourism

Leisure can be divided into universal factors and situational factors. Universal factors mainly include personal psychological factors, physiological factors, and socio-economic factors, while situational factors mainly include environmental and social resource aspects (Chang and Gibson, 2015). The universal factors are closely related to the characteristics of the individuals themselves. From a psychological perspective, the influencing factors of leisure may include personality, needs, motivation, attitude, and other psychological characteristics. For example, Diener et al. (2003) found that people with different personality characteristics have different leisure preferences. Based on Cognitive Appraisal Theory (CAT), internal factors (e.g., individual goals, motivations, expectations, needs) and external factors (e.g., identification with particular societies and groups, interactions, and policies) have an influence on individual behavior (Klaus and Scherer, 1993).

As a result of the combined effect of internal and external factors, individuals will experience different emotions in specific situations, and these emotions directly affect their behavior (Cohen et al., 1991). As far as tourism behavior is concerned, the psychology of tourists has been identified as playing an important role between the various influencing factors and tourists' behavior. Furthermore, tourists' travel motivations and travel preferences are often associated with emotions. The type and intensity of the travel motivation can have an impact on emotions experienced during traveling. Emotions are relevant in all stages of tourist behavior (Prayag et al., 2013). In the pre-tourism desire arousing stage, emotions are fundamental in evoking tourism motivation and tourism involvement. During the stage of decision-making and the journey, the emotional state of tourists at different time periods and their tourism behavior are constantly interacting with each other (Scott, 2020). The value of tourism is largely assessed through emotions. For tourists, the ultimate value of all tourism behaviors is to obtain positive emotional enjoyment and delight from their travel experiences (Hosany et al., 2020). The various factors associated with the external or internal environment generate the emotional connection between tourist destinations and tourists, which plays a key role in understanding tourist behavior (Liu et al., 2016).

Physiological factors can also influence leisure tourism behavior. For example, individuals with stronger health and enjoyment motives, are more likely to participate in leisure activities. Research from a socio-economic perspective pays attention to people's external characteristics such as economic income, occupation category, age group, family structure, gender, marital status and so on. For example, Zhao et al. (2013) conducted an empirical study on the leisure behavior of urban residents in Nanjing, and the results showed that social and economic attributes such as age, education background and income were key factors affecting residents' leisure activities and their choice of travel modes. Jiang et al. (2011) found that children in a family affect the frequency and type of leisure activities.

Situational factors are different from universal factors in that they are mainly associated with environmental and social resource aspects including interpersonal factors and structural factors. Interpersonal factors are generated in the process of communication and interaction between leisure participants and others, such as the opinions of peers, family members and friends. Structural factors often play a mediating role between leisure preference and leisure participation, including appropriate access to leisure facilities, leisure opportunities, social and cultural environment, overall economic development level of society, and relevant policies (Dai et al., 2019). According to the hierarchical model of leisure constraints developed by Crawford et al. (1991), individual intrinsic factors are the most important factors that directly affect leisure participation, while interpersonal factors and structural factors are secondary to individual intrinsic factors.

Leisure tourism is different from leisure. The latter is not linked to a spatial location, however leisure tourism is often associated with distant places. The influencing factors of leisure are mainly associated with individual, interpersonal, and social aspects. However, the main factors of leisure tourism also include considerations of what tourism destination to travel to and transitional factors between the tourism destination and the tourist's origin (Werner and Andreas, 2006). The products, facilities, marketing, image, and tourism agencies of a particular tourism destination will also have an impact on an individual's perception of leisure tourism.

The above literature review can be used as a reference for research on the factors influencing leisure tourism of the visually impaired. However, the factors influencing leisure tourism of the visually impaired also have their particularities and differences. While their visual senses are impaired due to congenital or acquired factors (Guo, 2005), they can perceive the world by hearing, smell, taste and touch (Darcy and Daruwalla, 1999). There is a lack of research on the factors influencing leisure tourism of visually impaired individuals. This is a gap this study is attempting to fill by identifying the factors affecting the leisure tourism behavior of visually impaired individuals and by proposing a theoretical framework based on the identified factors.

## Research Methods

### Data Collection

This exploratory study uses a qualitative research design to explore the factors influencing the leisure tourism behavior of visually impaired individuals. Semi-structured in-depth interviews were used to obtain detailed situational information of visually impaired research participants. The interviews took place from July to August 2020. Considering the spatial dispersion and confidentiality of the research participants, as well as the sensitivity of the research topic, this study adopted a purposive sampling strategy. The official WeChat account of a professional travel agency for the visually impaired was used to recruit potential research participants. Interviewees were recruited from all over China; hence data collection was carried out through one-on-one in-depth telephone interviews. The question schedule used during the interviews was developed based on the existing literature and research needs. Before the formal interview, the question schedule was pilot tested with three visually impaired people and appropriate adjustments were made to reduce any ambiguities. Key interview questions included: What do you do in your spare time? Have you previously participated in a leisure tour? What are the reasons for your wish to travel? What factors affect your travel behavior? The semi-structured questions worked well in providing the research participants with the opportunity to express themselves, while further prompts were used to trigger in-depth discussions on the research topic.

In this study, saturation was achieved after interviewing 21 participants, and the final 5 interviews did not provide any new insights. Interviews lasted about 40–60 min, and all interviews were recorded with the consent of the participants. After the interviews, the recordings were transcribed word-for-word into Chinese text for analysis.

The profile of the research participants is shown in Table 1. Of the 26 participants, 16 were men and 10 were women. Sixteen were completely blind and 10 were severely amblyopic. More than half of the research participants have a high school or technical secondary school education as their highest education, while only six had a university degree. In terms of employment, there were 11 research participants who were engaged in non-massage related jobs, including writing, music, software promotion, artificial intelligence, audio novel recording, finance, disabled persons' service center and other employment, breaking the stereotype of “the visually impaired can only do massage.”

**Table 1:** Profile of interview participants (*N* = 26).

**Items**	***N***	**%**
**Gender**		
Male	16	61.5
Female	10	38.5
**Age**		
20–30	12	46.2
30–40	10	38.5
40–50	4	15.4
**Vision**		
Serious amblyopia	10	38.5
Blindness	16	61.8
**Annual income/10,000 Yuan**		
≤ 1	3	11.5
1–5	5	19.2
5–10	9	34.6
10–15	5	19.2
15–20	3	11.5
20–25	1	3.8
**Education level**		
Primary school	1	3.8
Junior high school	5	19.2
High school or technical secondary school	14	53.8
University or college	6	23.1
**Employee type**		
The massage industry	15	57.7
Literary worker	1	3.8
Voice workers	3	11.5
Internet-related	1	3.8
Finance	2	7.7
Disabled persons' service center	1	3.8
Students	3	11.5

### Data Analysis

Analysis involved identifying and coding any emergent themes using a structured Grounded Theory approach (Luborsky, 1994). With the help of the NVivo software (version 12, QSR), the following types of coding were conducted: open coding, axial coding and selective coding (Strauss and Corbin, 1998), to explore and analyze the main themes in the data, and to construct a theoretical framework of the factors affecting leisure tourism behaviors of visually impaired individuals.

In the stage of open coding, the interview transcripts were categorized into common themes. This involved reading and sorting the transcripts to establish free nodes of theme-related original statements, which resulted in 274 original categories. There were some semantic crossovers, and semantic repetitions. To reduce the crossovers and repetitions, the 274 original categories were refined undergoing a process of repeated comparison and integration. This process resulted in 75 categories with independent connotations.

In the axial coding stage, the categories were analyzed for their thematic ideas and relationships to one another, which resulted in 21 categories. These categories included: tourist information, management support, service level, tourism products, tourism preference, tourism motivation, personality, emotional state, knowledge background, economic status, physical condition, spare time, perceived stress, social environment, policy support, visually impaired tourism organizations, volunteer services, family support, traffic and transport accessibility, buildings and facilities accessibility, information accessibility.

Finally, the selective coding process involved further clarifying and refining of any relationships and organizing the identified categories around a central explanatory concept. This resulted in six core themes being identified, including tourism products and services, personal psychological factors, personal socio-economic factors, social support, community support, and barrier-free environments.

**Figure 1:**
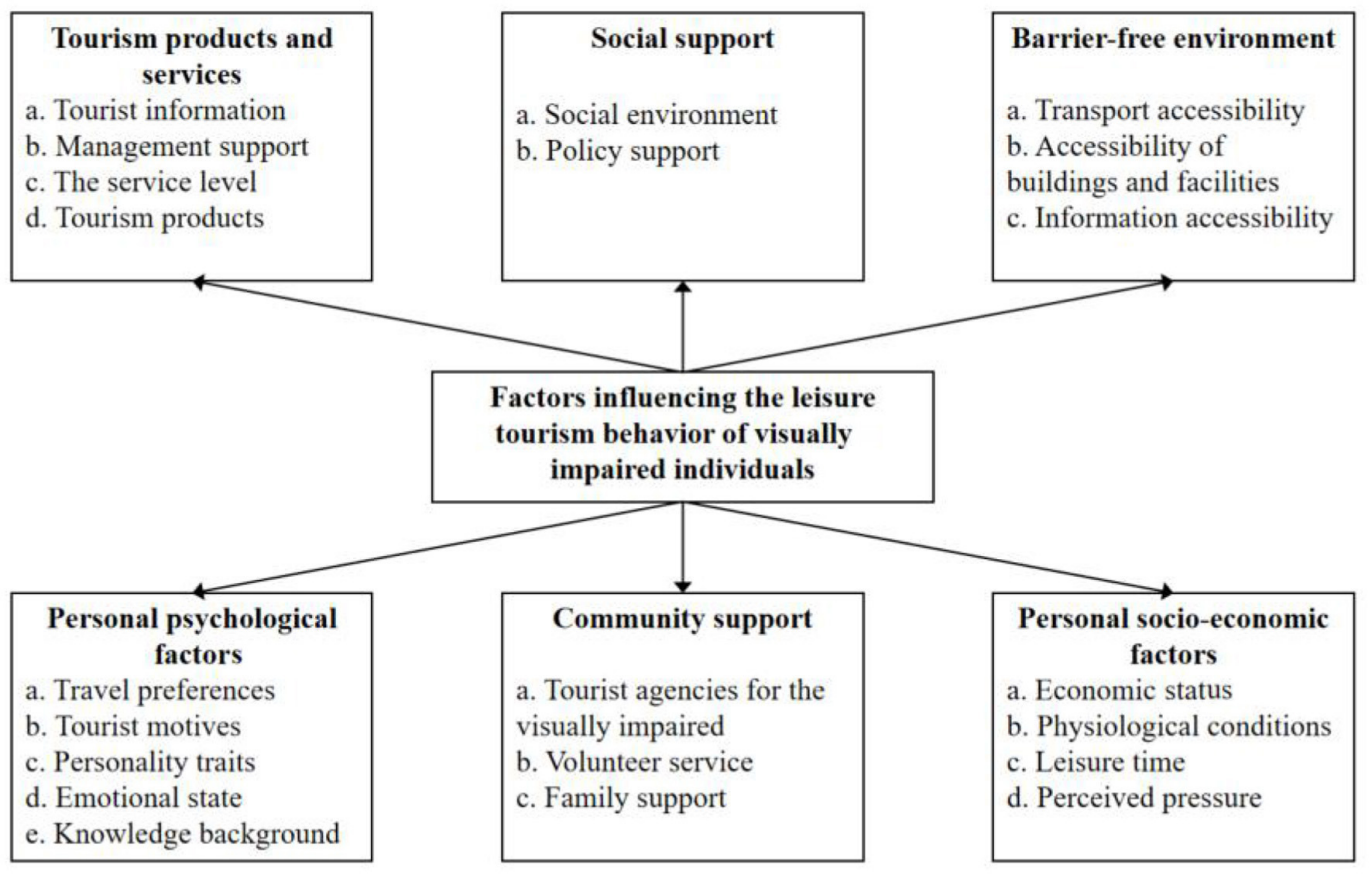
Factors influencing the leisure tourism behavior of visually impaired individuals.

Finally, to ensure the quality of the coding process and interpretation, the coded results were checked by both the research members and the interviewees (Cho and Trent, 2006). Based on the above analysis, the interrelationships between categories have been clearly defined. The six main themes and their corresponding categories were used as the foundation to construct a theoretical framework which outlines the factors influencing leisure tourism behaviors for visually impaired persons, as shown in Figure 1.

## Findings

**Figure 2:**
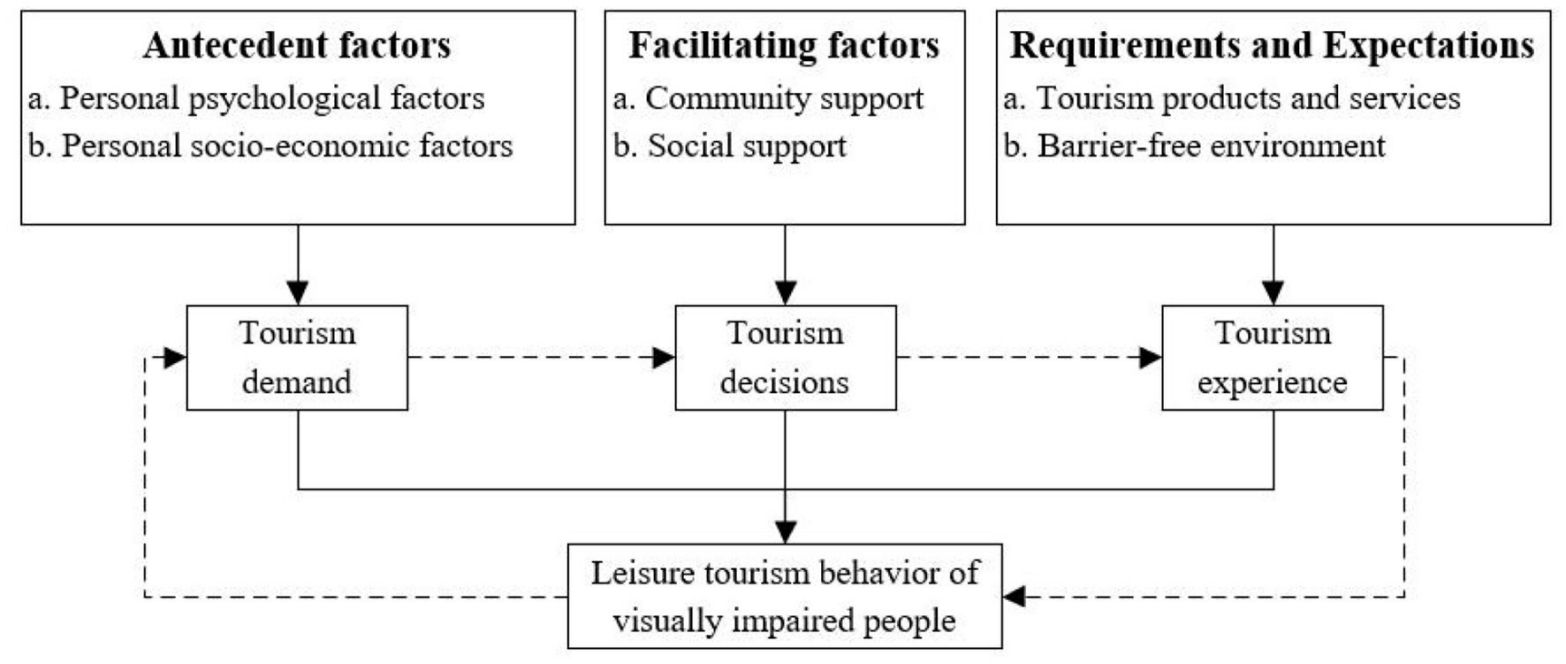
Path model showing the factors influencing visually impaired individuals during the various tourism stages.

As a result of the content analysis of the transcribed text, the travel process of the visually impaired can be divided into three parts: travel desires, travel decision-making, and travel behavior resulting from the different psychological states of tourists at different travel stages. By reviewing the descriptions of the interview participants, it can be ascertained that most of the influencing factors identified in this study on the travel behavior of visually impaired tourists are associated with emotions and travel intentions. The six identified factors including personal psychological states, personal socio-economic situations, social support, community support, tourism products and services, and barrier-free environments can be classified into antecedent factors, facilitating factors, requirement and expectations factors (as shown in Figure 2).

### Antecedent Factors

The antecedent factors are comprised of subjective factors and objective factors. These consider personal psychological factors such as travel motives and preferences as well as personal socio-economic factors including employment and disposable income. The visually impaired are motivated by subjective factors (i.e., travel preference, personality traits, etc.), and have a desire to travel. However, participation in tourism experiences is warranted only when certain objective conditions are in place (i.e., appropriate socio-economic factors).

Table 1 showed that the massage industry is the main employment of the visually impaired respondents in this study: “My job is providing massage services to guests in a small room day by day. When I finish working, I always stay at home. My life is so boring” (R15). This quote exemplifies that research participants longed to escape from their everyday lives through tourism experiences which contributes to their social and psychological well-being. Tourism has become an effective way for the visually impaired to satisfy their desires. Moreover, due to the limited choice of leisure options available for the visually impaired, tourism has become a preferred way to spend their spare time: “For me, traveling is a good way to spend my leisure time. I haven't found a better way to spend my leisure time than traveling, and for me, I have very few options for leisure” (R7). Visually impaired research participants who frequently participate in travel experiences, recognize tourism experiences as an opportunity to improve their personal abilities which creates further desire for tourism: “tourism is actually a process to train comprehensive ability, such as problem-solving ability, psychological adjustment ability. During the trip, there are always unexpected incidents that happen, which can provide me with these opportunities to grow, and to improve myself” (R5).

The interaction between subjective factors and objective factors stimulates tourism demand (Torgeir et al., 2018), i.e., having appropriate leisure time and disposable personal income enables the vision impaired to travel. One respondent said “If time and money allow it, I will travel as much as possible, but money and time are necessary (R12).” General health and physical fitness also affect whether visually impaired respondents are able to participate in leisure tourism: “The reasons that hinder me from going out are that I am tired and I have nobody who I could travel with. But, as long as I have the energy to travel and someone can take me, I will go out” (R19).

### Facilitating Factors

Antecedent factors are involved in most peoples' travel considerations. However, for the visually impaired, there are further considerations which are based on facilitating factors including the community and social support. Community support mainly involves the notion of travel companions who help the visually impaired to navigate their travels. For example, R4 mentioned: “My completely blind friend also wants to travel, but his family does not want to accompany him, so it is difficult for him to travel. My family is quite supportive and sometimes urges me to travel with me” (R4). Volunteer services during the whole trip are also an important facilitating factor in the travel decision of visually impaired people, as they rely on them for necessary assistance during their travel, particularly for individuals with total blindness. If their family and friends cannot accompany them on their travels, and no volunteers are available to help during the trip, then they will not be able to realize their desire to travel. One respondent and his wife are both blind and recognize the importance of the volunteers: “If my wife and I are the only two people to travel, it is definitely not possible. It is very important to have volunteers to help us solve some troubles during the trip.” If there were more professional tour organizers that provided travel services for the visually impaired, then that would be a preferred choice for many visually impaired travelers. For example, R3 mentioned “I only got to know the ZS tourism team by chance, but I would try and join in every travel route they offer as long as I had time. Because their team is very helpful with vision impaired people, and all their itineraries are determined according to the needs of the visually impaired, and there are volunteers to accompany us throughout the journey, so it is very convenient to travel with them.” Social support mainly refers to aspects such as policy support and attitudes toward visually impaired individuals by the public. R10 mentioned: “Disability passes are helpful in relieving some of the financial pressure, and it's even better if we can get discounts on things like sightseeing buses.” A supportive social environment was important for R4: “Actually I don't have any particular idea where to go, but no matter where, as long as the people there have a good attitude I'm willing to have a try.”

### Requirements and Expectations Influencing the Tourism Behaviors

Once the vision impaired tourists arrive at the tourism destination, the availability of appropriate tourism products and services, and barrier-free environments become the key elements to affect their behavior and their psychological and emotional well-being. One of the main considerations for the visually impaired with any tourism product and service is the safety of products, as well as the professionalism of any services offered. The participation in tourism products is a core aspect for vision impaired tourists, and the degree of participation directly affects their behavior and satisfaction. The higher the degree of participation, the better the satisfaction (Chen et al., 2020). Hearing, touch and taste are the main ways for the visually impaired to participate in tourism activities (Clawson and Knetcsh, 1969). The majority of respondents (81%) mentioned the importance of taste as part of their travel experience. Taste was particularly important for this respondent: “No matter how interesting an experience is, what I can feel is limited. Even if I touch those things, I can't feel anything. I think it's better to feel through tasting with your mouth. That's really participating” (R10). Another respondent emphasized the influence of hearing and touch on the mood after a tour: “I personally think that hearing and touch have even more important effects on the tourist experience, for example, listening to the interpretation and touching exhibits, cannot only contribute to the physical experience but also bring spiritual enjoyment. If I travel to a place where there is nothing to touch, I feel a little bit of regret” (R12). Personal safety is another important aspect during their stay in the destination, as R26 mentioned: “I think safety comes first. Climbing a mountain is risky for me. If there is no guardrail and steps are not skid proof, I certainly dare not try it.” In addition, due to the lack of awareness about the specific needs of visually impaired tourists, their participation in tourism activities is often limited, which is typically a negative emotional point in the process of the visually impaired tourism. For example R13 mentioned: “When we travel, many scenic spots and recreational facilities do not allow vision impaired people to enter, which is the most frequent conflict resulting in negative emotions. Some places won't let us in, no matter how much we negotiate, even if we come in the company of others. If vision impaired people are not allowed in, why not post a notice online in advance? We traveled a long way to get to this scenic spot, and when we arrived, we were told we are not allowed in, which made us very depressed” (R13).

For the visually impaired, accessibility is an important expectation factor. The tourism behavior of the visually impaired is often seriously restricted by the environment (Kim and Seo, 2020), however the needs of the visually impaired for a barrier-free environment at the destination are frequently not met. In designing barrier-free environments, facility accessibility refers to the appropriate design of blind crossing tracks, barrier-free elevators, and barrier-free traffic lights. In this regards, R16 said: “I have been to many places where the accessibility is not good, and it is common for me to have some bumps. My aunt told me that they have good accessibility in Hong Kong, for example, there will be voice prompts at the traffic lights. I am looking forward to traveling there as soon as possible.” Another accessibility consideration at destination level is about creating awareness in the community of the special needs of visually impaired people and their tourism behavior. R14 pointed out: “Society doesn't understand us very well. Sometimes the scenic spots are crowded, and when we walk, we will bump into others, and they will be unhappy. Sometimes we need to ask for directions, and some people can be cold.”

## Discussion and Conclusions

This research contributes to the development of tourism theory focused on visually impaired individuals by identifying the factors influencing their travel behavior. The study also contributes to tourism practice because it supports the viewpoint that the vision impaired are an emerging tourism market segment which is important and has immense development potential based on empirical research. Furthermore, the findings may help tourism-related companies to better understand the travel needs of visually impaired groups as well as the factors influencing their travel behavior. There are practical implications for tourism managers in programming and optimizing tourism products and services that better serve visually impaired tourists, and further improve the social well-being of visually impaired groups. In a broader view, this may also promote a more harmonious society, due to an increased awareness of the visually impaired and their specific needs when traveling.

Apart from practical implications, the study also makes a theoretical contribution by proposing a new theoretical framework which outlines the major factors influencing the leisure tourism behavior of visually impaired individuals. It not only provides a new theoretical model focusing on the leisure tourism behavior of visually impaired travelers, but also offers a theoretical basis for future empirical research studies and scale development of factors influencing leisure tourism for the visually impaired. Furthermore, this study proposed a specific definition of leisure tourism for visually impaired individuals.

Specifically, the six dimensions of tourism products and services, individual psychological factors, social support, organizational support, individual socio-economic states and barrier-free environments and the 21 sub-categories summarized from this research are an independent system that has similarities and differences with the influencing factor system of the tourism behavior of sighted people. Five of the six identified factors outlined in Figure 1 will also influence the leisure tourism behavior of sighted tourists except for the need of a barrier-free environment and its accessibility dimensions. However, a closer look at the specific factors and their various concepts reveals significant differences between visually impaired travelers and sighted travelers. Compared with sighted travelers, the factor most likely to affect the leisure tourism behavior of the visually impaired is their reliance on other senses such as hearing, touch, taste and smell. To create a quality leisure tourism experience for the visually impaired, such experiences should combine landscape explanations, tactile design elements, and physical participation, while also making full use of imagination to produce vivid pictures and feelings in their minds and hearts (Liu et al., 2018). We learnt from the interviews that many visually impaired people are not limited by their visual impairment. When they travel, they have family members or volunteers as travel companions to provide them with detailed landscape explanations or meet their desire to touch objects by way of physical simulation. However, the interviews also highlighted that many visually impaired tourists are unable to meet their needs for landscape explanation and touch at various attractions or destinations. Tour guides in scenic spots mainly serve sighted tourists. When visitors to an attraction happen to be visually impaired, few tour guides adjust their explanations according to the specific needs of visually impaired individuals. Moreover, China's various attractions and scenic spots do not provide special facilities for visually impaired visitors. Even if a visually impaired person buys a ticket and brings an accompanying person with them, they will often be denied access to some tourist activities, such as ships, cable cars, glass walkways, rafting, and scuba diving, because the tourism operators are not willing to take any risks.

There are also differences in the motivational factors between visually impaired and sighted tourists. While seeking a pleasant feeling is a basic motivation for visually impaired tourists, they also seek to fulfill higher-level motivations such as perfecting one's own character and improving one's own ability, as a way to reduce the pressures on their family. Another important motivational aspect was being able to get away from their usual environment through tourism and to increase contact with other people. Increased contact with other people from society can be helpful in breaking down social stereotypes associated with visually impaired individuals and to promote their integration and the enhanced development of products and services targeting them.

Physical obstacles linked to visual impairment are often perceived more serious than psychological obstacles. Therefore, travel companions/escorts, and tour organizers focusing on the visually impaired, and accessibility issues are contributing factors in making traveling a more pleasant experience for the visually impaired. Finding your way, crossing the street, taking an elevator, lodging, dining/eating, taking public transportation, experiencing the sights - all of these actions require external assistance. There is a reliance on volunteers, family and friends and other travel companions to provide guidance for the visually impaired during their leisure tourism experiences. For example, it is difficult to tell the location of dishes when eating or the room furnishings when staying in a hotel. When family members and friends are unable to accompany the visually impaired travelers, tour organizers can provide professional tourism products and travel services to them. When people who are not completely blind travel by themselves, their requirements should also be considered, particularly in relations to their safety and convenience through an accessible environment.

The study reports the following limitations. All interviewees who participated had high levels of travel experience. The sample may not be representative of vision impaired people who have no prior travel experience. The reasons as to why some vision impaired individuals choose not to participate in tourism may provide further important influencing factors of their leisure tourism behavior. Furthermore, most of the interviewees were positive and optimistic, which may represent a particular type of personality among the sample of vision impaired people who participated in this study. Hence, future studies may investigate visually impaired individuals who do not have any prior travel experience and who do not participate in leisure tourism activities, as a helpful way to discover the barriers which prevent them from traveling. Furthermore, the COVID-19 pandemic has had unprecedented impacts on the global tourism industry. Several countries have implemented major measures for the effective prevention and control of further transmission, and domestic tourism is slowly starting to recover. However, it is unclear how the COVID-19 pandemic affected visually impaired individuals, which will make a valuable contribution in future studies.

## Data Availability Statement

The original contributions presented in the study are included in the article/supplementary material, further inquiries can be directed to the corresponding author/s.

## Ethics Statement

The studies involving human participants were reviewed and approved by Zhejiang Gongshang University. The ethics committee waived the requirement of written informed consent for participation.

## Author Contributions

GQ: conceptualization, methodology, validation, formal analysis, investigation, resources, data curation, and writing-original draft preparation. JZ: writing–review and editing, methodology, and validation. AP: writing–review and editing, supervision, and project administration. NC: conceptualization and writing–review and editing. All authors contributed to the article and approved the submitted version.

## Conflict of Interest

The authors declare that the research was conducted in the absence of any commercial or financial relationships that could be construed as a potential conflict of interest.
